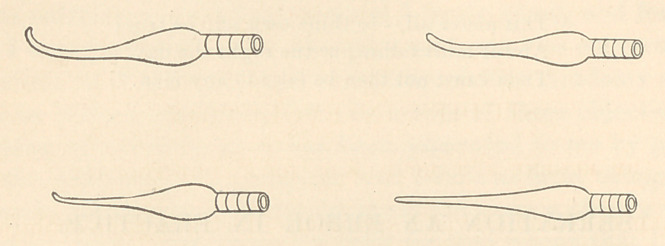# Serration an Error in Practice

**Published:** 1898-06

**Authors:** J. W. Worcester

**Affiliations:** Middletown, N. Y.


### SERRATION AN ERROR IN PRACTICE.

BY J. W. WORCESTER, M.D., MIDDLETOWN, N. Y.

Having read with much interest your editorial on "The Evolution and Abuse of the Serration" in the March, 1897, issue of the International Dental Journal at the time of its receipt, I have at the present moment again gone over the subject for the purpose of giving you my opinion in the matter (which I hope will be acceptable and of benefit to the profession), not on the "evolution" of the serration, but on the "abuse" of it.

I came to the conclusion some years ago that serrated instruments were a *great mistake* (except with the most careful and painstaking operator), and therefore gave up their use.

My reasons for so doing were principally confined to the fact that the serrations were the cause of so many leaky fillings through the abrasion or chipping off of the tooth-substance from the walls of the cavity during the operation, and which became more or less incorporated and condensed with the gold, despite the use of the chip-blower.

This bone-dust, being a perishable substance and continuing to the edges of the cavity in the filling operation, as it invariably is with these instruments, was without doubt, and is yet to those using them, the principal cause of leaky fillings, especially with the beginners and also with many of the elders.

The acid condition so often present around the teeth furnishes means for making a solution of this confined bone-dust, with the result familiar to all.

To overcome the difficulty and make an absolutely tight plug, I adopted the method of using the smooth point, not a burnished smooth point, but one that is rubbed over frequently with a spun-glass burnisher or a medium-grit emery disk, although I use both constantly. These keep the steel point clean and bright, and with the electric mallet, which I use (and which should be preferably used with these instruments), I can weld the gold perfectly solid and intact, and will not injure or abrade the walls of the cavity in the least.

You can mallet around at the junction of the gold and the tooth with these instruments, and the result is a tight joint with *no* bone-dust intermixed.

The instruments I use are round, either straight or curved, with rounded points, such as I here illustrate, and will be found perfectly practicable in all cases for gold work, using several sizes.

I invariably use Watts's crystal gold cubes (which can be welded perfectly with these instruments), without annealing, until I get near the surface, when, with the use of annealed foil pellets of a very light form (smallest size), I bring the filling to a finish and polish off.

My method of preparing the cavities is to first grind or disk away all frail walls around the cavity to a self-cleansing surface and polish. Then I proceed to excavate the cavity with just enough undercut to hold the filling, leaving *no sharp* angles therein, square across at the cervical walls, and round off the edges of the cavity (as it joins the previously polished surface) with a fine finishing bur or fine office-made emery points. The rubber dam is applied and ligated to the teeth and the moisture removed with absorbent paper, then wiped out with sulphuric ether, and lastly with a solution of bichloride of mercury in chloroform one to one thousand. The cavity is now in excellent condition to fill, and I carry this out in the preparation of all cavities for whatever kind of filling.

After the cavities are filled with a slight overhang I disk down (to the original polished surface previous to filling) and continue to polish until impossible to do so more.

These fillings I find give good service. I would like to see the profession generally take as much pains and do better dental work, and abolish the pernicious habit of using serrated instruments as I consider them an abomination and a curse in the hands of most operators. Let the law be laid down that the use of simple, smooth, scratch-points, as applied to gold work, is the sure road to success and a glorious beneficence to humanity in the field of dentistry. If there is any improvement on these methods I would like to see it.

## Figures and Tables

**Figure f1:**